# The association between the pre-pregnancy vaginal microbiome and time-to-pregnancy: a Chinese pregnancy-planning cohort study

**DOI:** 10.1186/s12916-022-02437-7

**Published:** 2022-08-01

**Authors:** Xiang Hong, Jun Zhao, Jiechen Yin, Fanqi Zhao, Wei Wang, Xiaoling Ding, Hong Yu, Xu Ma, Bei Wang

**Affiliations:** 1 Key Laboratory of Environmental Medicine and Engineering of Ministry of Education, Department of Epidemiology and Health Statistics, School of Public Health, Southeast University, No. 87 Dingjiaqiao Rd., Gulou District, Nanjing, Jiangsu China; 2 National Research Institute for Family Planning, Beijing, China; 3 National Human Genetic Resources Center, Beijing, China; 4 Maternal and Child Health Center of Gulou District, Nanjing, China; 5 Department of Obstetrics and Gynecology, Zhong Da Hospital, Southeast University, Nanjing, China

**Keywords:** Fecundability, Time-to-pregnancy, Vaginal microbiome, *Lactobacillus*, Cohort study

## Abstract

**Background:**

Although sexually transmitted infections are regarded as the main cause of tubal infertility, the association between the common vaginal microbiome and female fecundability has yet to be determined. The objective of this study was to find convincing evidence relating to the impact of the vaginal bacterial structure on the fecundability of women planning pregnancy.

**Methods:**

We recruited women who took part in the Free Pre-pregnancy Health Examination Project from 13 June 2018 to 31 October 2018 (*n* = 89, phase I) and from 1 November 2018 to 30 May 2020 (*n* = 389, phase II). We collected pre-pregnancy vaginal swabs from each subject; then, we followed up each subject to acquire the pregnancy-planning outcome in 1 year. In phase I, 16S rRNA gene sequencing was performed to investigate the vaginal bacterial content between the pregnancy and non-pregnancy groups. These findings were verified in phase II by applying a quantitative real-time polymerase chain reaction for the measurement of the absolute abundance of specific species. Cox models were used to estimate fecundability ratios (FR) for each vaginal microbiome type.

**Results:**

In phase I, 59.6% (53/89) of women became pregnant within 1 year. The principal coordinate analysis showed that the pre-pregnancy vaginal microbial community structures of the pregnant and non-pregnant groups were significantly different (PERMANOVA test, *R*^2^ = 0.025, *P* = 0.049). The abundance of the genus *Lactobacillus* in the pregnancy group was higher than that of the non-pregnant group (linear discriminant analysis effect size (LDA) > 4.0). The abundance of the genus *Gardnerella* in the non-pregnant group was higher than those in the pregnant group (LDA > 4.0). In phase II, female fecundability increased with higher absolute loads of *Lactobacillus gasseri* (quartile Q4 vs Q1, FR = 1.71, 95%CI 1.02–2.87) but decreased with higher absolute loads of *Fannyhessea vaginae* (Q4 vs Q1, FR = 0.62, 95%CI 0.38–1.00). Clustering analysis showed that the vaginal microbiome of type D (characterized by a higher abundance of *Lactobacillus iners*, a lower abundance of *Lactobacillus crispatus* and *Lactobacillus gassri*) was associated with a 55% reduction of fecundability (FR = 0.45, 95%CI 0.26–0.76) compared with type A (featuring three *Lactobacillus* species, low *Gardnerella vaginalis* and *Fannyhessea vaginae* abundance).

**Conclusions:**

This cohort study demonstrated an association between the pre-pregnancy vaginal microbiome and female fecundability. A vaginal microbiome characterized by a higher abundance of *L. iners* and lower abundances of *L. crispatus* and *L. gasseri* appeared to be associated with a lower fecundability. Further research now needs to confirm whether manipulation of the vaginal microenvironment might improve human fecundability.

**Supplementary Information:**

The online version contains supplementary material available at 10.1186/s12916-022-02437-7.

## Background

Infertility has become a severe public health problem; more than 186 million people suffer from infertility worldwide [1]. A reduction of human fecundability not only affects the physical and mental health of pregnancy-planning couples, but also results in a general trend towards an aging population [1, 2]. Despite many efforts to explore the factors that influence human fecundability, there are still many unanswered questions. Previous case-control studies showed that there were some potential differences between infertile and fertile women with regard to the vaginal microbiota and that a low*-Lactobacillus* vaginal microbiome appeared to be a risk factor for infertility [3, 4]. However, it has proven difficult to determine the causal relationship between these factors, largely because vaginal sampling is not performed before infertility diagnosis. On this basis, our previous study used a prospective design and the Chinese National Free Pre-conception Check-up Project database to illustrate that a poor vaginal microenvironment was associated with a longer time-to-pregnancy (TTP) in normal healthy women [5]. In another study, Lokken et al. found that women with bacterial vaginosis (BV) may be at an increased risk of sub-fecundity in a Kenyan pregnancy-planning cohort [6].

However, traditional microscopic examination cannot reveal the structural characteristics of the vaginal microbiome [7], thus limiting the study of fertility-related vaginal species. Common vaginal *Lactobacillus* species include *Lactobacillus crispatus* (*L. crispatus*), *Lactobacillus iners* (*L. iners*), and *Lactobacillus gasseri* (*L. gasseri*). Different vaginal *Lactobacillus* species have been found to exert different health effects over recent years; for example, when the microbiota is dominated by *L. iners*, there is a higher likelihood of a shift towards dysbiosis [8, 9]. However, routine tests cannot distinguish different *Lactobacillus* species, and there is no evidence to show which *Lactobacillus* is most beneficial for female fecundability from prospective studies. Furthermore, recent studies have identified substantial divergences in the vaginal microbiome structure between healthy individuals from different races and ethnicity [10]. The incidences of vaginal communities with several non-*Lactobacillus* species gradually increase from European to Asian to African populations [11]. Therefore, it is vital that we investigate the association between female fecundability and the vaginal microbiome in Chinese cohorts. In the present study, we recruited a pregnancy-planning cohort of subjects to investigate the association between female fecundability and the vaginal microbiome, thus providing a new concept for female fertility intervention strategies.

## Methods

### Study population

Between 13 June 2018 and 30 May 2020, all couples who took part in the Free Pre-pregnancy Health Examination Project in the Maternal and Child Center of Gulou district in Nanjing, China, were invited to join this study cohort. The inclusion criteria were as follows: (1) according to the Chinese legal marriageable age, the female needed to be older than 20 years, and the male needed to be older than 22 years, and all of them should be less than 49 years old, and (2) couples who reported that they were ready to become pregnant. The exclusion criteria were as follows: (1) females who had been pregnant when taking part in the project; (2) either partner had been diagnosed with a medical condition unsuitable for pregnancy, including uterine malformation, testicular loss, and *Treponema pallidum* infection; (3) the women who had some diseases related to fertility, such as endometritis, polycystic ovarian syndrome, uterine fibroids, and pelvic inflammatory disease; (4) women who refused to provide vaginal swabs; (5) women who had used antibiotics in the previous 2 weeks; and (6) women who were lost to follow-up (data available for only baseline, without one visit).

**Fig. 1:**
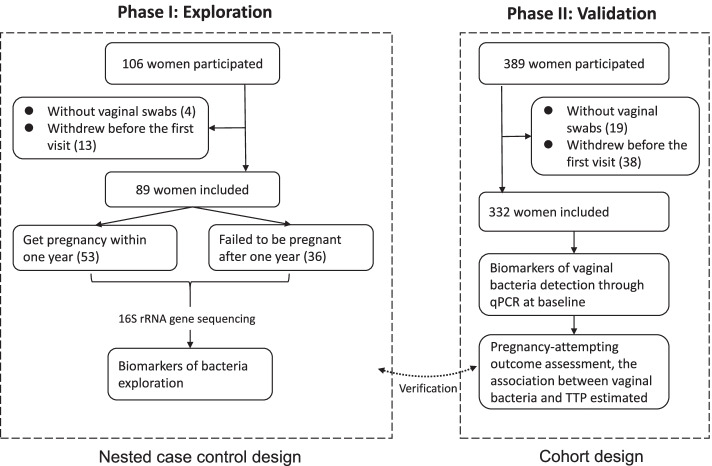
Study design and participants

This study was divided into two phases. Phase I was conducted from 13 June 2018 to 31 October 2018; 106 women participated in this phase. This phase featured a nested case-control design. All participants were divided into pregnant or non-pregnant groups according to pregnancy outcomes after 1 year of participation. The potential biomarkers for bacteria that were identified in phase I were then detected in phase II, and their associations with TTP were verified by a cohort design. Phase II was conducted from 1 November 2018 to 30 May 2020. In total, 500 women were invited, and 495 women signed the informed consent; 23 women refused to provide vaginal swabs because of a menstrual period, and 51 women withdrew without the first visit. The final study included 89 women in phase I and 332 women in phase II. Further details are shown in Fig. 1.

Sample size estimations were performed when the research protocol was first designed (further details are provided in Additional file 1 [12]). All participants signed an informed consent form, and the study was approved by the Ethics Committee of Zhongda Hospital (Reference: 2018ZDSYLL116-P01).

### Acquisition of data for covariate analysis

At baseline, we performed a unified epidemiological survey for every female so that we could collate their age, the age difference within couples, educational level (high school and below/higher education and above), occupation (workers/office clerk/others), pregnancy history (yes/no), and menstrual status (regular or not). A regular menstrual cycle was defined as a cycle length of 21–35 days [13]. All data were acquired by one professional nurse to ensure that the information was credible.

### Outcome assessment

All females would be contacted by the medical staff (by telephone) every 3 months. The main outcome was clinical pregnancy, as self-reported by the subjects; this needed to be confirmed by a pelvic ultrasound scan in the hospital. TTP was the interval between the dates of the last menstrual period (LMP) obtained at follow-up and before conception (pregnant within 1 year) or the last follow-up call (if not pregnant). TTP in months was calculated by TTP in days/30. The TTP in cycles was calculated by TTP in days/average length of the menstrual cycle. These indices were all round up to an integer.

### Vaginal swab collection and nucleic acid sequencing

Women were placed in a lithotomy position under standard operating procedures; then, gynecologists obtained two vaginal swabs with the aid of a sterile speculum. Swabs were rotated three times on the vaginal fornix to uniformly scrape any discharge and were transported to the laboratory within 4 h. Vaginal cleanliness was graded via microscopic examination of cervical smears. Grades I and II were regarded as normal while grades III and IV were regarded as disordered; these gradings were in accordance with the Chinese standards [14]. The second swab was stored in a dry tube at − 80 °C to await nucleic acid extraction. The detailed procedures for DNA extraction and 16S rRNA gene sequencing are described in Additional file 1. In brief, the swabs were eluted with PBS buffer and the TIANamp Bacterial DNA Kit (Tiangen Biochemical Technology, Beijing, China) as used to extract and purify nucleic acids. The V3–V4 region of the 16S rRNA gene was amplified and sequenced on an Illumina HiSeq 2500 platform (Beijing Biomarker Technologies Co. Ltd., Beijing, China). The raw sequencing data is stored in the figshare platform [15]. Then, sequencing data were processed using a standard procedure (Additional file 1). Denoised sequences were clustered using USEARCH (version 10.0), and tags with ≥ 97% similarity were regarded as an operational taxonomic unit (OTU). Representative sequences were annotated through the National Center for Biotechnology Information (NCBI) dataset using the QIIME software (https://qiime2.org). The numbers of reads for each sample were normalized according to the sample with the least sequence. All bioinformatics analyses were completed on the Biomarker BioCloud platform (www.biocloud.org).

We used the QIIME2 software (http://qiime2.org/) to calculate α diversity for the vaginal microbiome, including Shannon, Simpson, Chao1, and ACE indices. These indices reflect the richness and diversity of the microbial community structure [16]. Based on the matrix of relative abundance of bacteria, we estimated the Jaccard Distance Index and then performed principal coordinate analysis (PCoA) to intuitively display different groups of the microbiome. Next, we performed a permutational multivariate analysis of variance (PERMANOVA) to test for statistical significance. Linear discriminant analysis (LDA) effect size (LEfSe) was used to identify potential biomarkers among different groups, which should meet *P* < 0.05, adjusted *P* < 0.01, and LDA > 4.0 [17]. The Benjamini-Hochberg (BH) method was used to adjust *P* values to minimize the false discovery rate when performing multiple comparisons.

### Assessment of absolute bacterial loads and the clustering of microbial communities

Quantitative real-time polymerase chain reaction (qPCR) was used to measure the absolute loads of specific vaginal bacteria, including *L. crispatus*, *L. gasseri*, *L. iners*, *Gardnerella vaginalis* (*G. vaginalis*), and *Fannyhessea vaginae* (*F. vaginae*, also called *Atopobium vaginae*). We used specific primers that had been verified by previous studies (further details are given in Additional file 2: Table S1 [18–21]). We used the NCBI Blast database to predict the amplified products, and specific plasmids were synthesized by Sangon Biotech Company (Shanghai, China). The copy number concentration of the plasmid was calculated using the following formula: copies/mL = 6.02 × 10^23^ × 10^−6^ × concentration (ng/μL)/(fragment length × 660). Then, 10-fold serial dilutions of the plasmid were prepared and subjected to qPCR to obtain a standard curve. In order to reduce variations in the total bacterial load from different swab samples, the copy number concentrations of the 16S V3–V4 region were also measured; these were then standardized to 1 × 10^10^ copies/mL for each sample. Under this condition, we measured the absolute abundance of another 5 species.

For each species, we calculated the absolute abundance *z*-score after the logarithmic transformation of the absolute loads. The clustering of microbial communities was explored with the *k*-means algorithm which minimizes the error inside the groups and maximizes the distance between clusters. We considered the Euclidean distance metric in our analysis and then tried to use the elbow method to determine the optimum number of clusters [22]. In this method, the slow-down point denotes the optimum number of clusters. Then, we compared the average *z*-score for specific species among different clusters using variance analysis.

### Statistical analysis

All data were uploaded into the EpiData (Version 3.1) software by two independent researchers. The analyses followed a defined approach that was determined before running the models. Continuous variables are described by the mean and standard deviation (SD) (normal distribution) or median and quartile if not distributed normally. The *t* test or the Kruskal-Wallis test was used to test for the differences between the groups. Categorical variables are described by frequency and percentage; the chi-squared test or Fisher’s exact test was used to compare the distribution between the groups. Missing data were imputed by the multivariate imputation chained equations (MICE) package in the R software [23]. We set up five imputed datasets; the main analysis results were aggregated with Rubin’s rule after appropriate transformation [24]. We performed analyses using the completed case dataset as sensitivity analysis.

Spearman coefficients were calculated to determine the correlation between two relative abundances of bacteria. The Kaplan-Meier (KM) method was used to calculate the cumulative pregnancy rates in different types of microbiomes, and the log-rank test was used to test the differences. Cox models were used to estimate the fecundability ratios (FRs) and their 95% confidence intervals (CIs) for different types of microbiome after adjusting for potential confounding factors. FR reflects the ratio of pregnancies among females with certain characteristics compared with the reference groups; thus, an FR < 1.0 implies a lower fecundability or a longer TTP. All of these analyses were carried out using the R software (version 4.1.0), and two-sided probability values of < 0.05 were deemed to be statistically significant.

## Results

### The vaginal microbiome and fecundability in phase I

In phase I, the mean age of the participants was 28.66 ± 3.14 years old; most women did not have a history of pregnancy (79/89, 88.76%). In total, 59.6% (53/89) of women achieved pregnancy within 1 year. A comparison of the baseline characteristics between the two groups (pregnant or non-pregnant) revealed that there were no significant differences in terms of the age difference within couples, educational level, occupation, history of pregnancy, and the regularity of menstruation (*P* > 0.05, Table 1), although the mean age of the pregnancy group was significantly lower than that of the non-pregnancy group (27.98 vs 29.52, *P* = 0.036).

**Table 1:** Baseline characteristics of cohort phases I and II

	Phase I		*P*	Phase II		*P*
	Non-pregnancy	Pregnancy		Non-pregnancy	Pregnancy	
*N*^a^	36	53		185	147	
Age, years, mean (SD)	29.52 (3.73)	27.98 (2.67)	0.036	29.95 (4.39)	28.94 (3.25)	0.021
Age difference with their couples, years, mean (SD)	2.21 (2.90)	1.10 (3.00)	0.091	1.29 (3.44)	1.32 (2.63)	0.943
Educational level						
High school and below	6 (16.7)	3 (5.7)	0.183	27 (14.6)	17 (11.6)	0.242
Higher education and above	30 (83.3)	50 (94.3)		158 (85.4)	130 (88.4)	
Occupation						
Workers	1 (3.0)	1 (2.0)	0.647	3 (1.6)	1 (0.7)	0.164
Office clerk	22 (66.7)	38 (76.0)		128 (69.2)	115 (78.2)	
Others	10 (30.3)	11 (22.0)		54 (29.2)	31 (21.1)	
Pregnancy history						
No	32 (88.9)	47 (90.4)	0.999^b^	130 (71.0)	113 (76.9)	0.285
Yes	4 (11.1)	5 (9.6)		53 (29.0)	34 (23.1)	
Regular menstruation						
Yes	28 (80.0)	40 (76.9)	0.939	166 (89.7)	119 (81.0)	0.034
No	7 (20.0)	12 (23.1)		19 (10.3)	1 (0.7)	

^a^In phase I, there are 6, 1, and 2 women who missed educational level, occupation, and menstruation information, respectively. In phase II, there were 2 and 27 women who miss pregnancy history and menstruation information, respectively

^b^Fisher’s exact test. SD, standard deviation

**Fig. 2:**
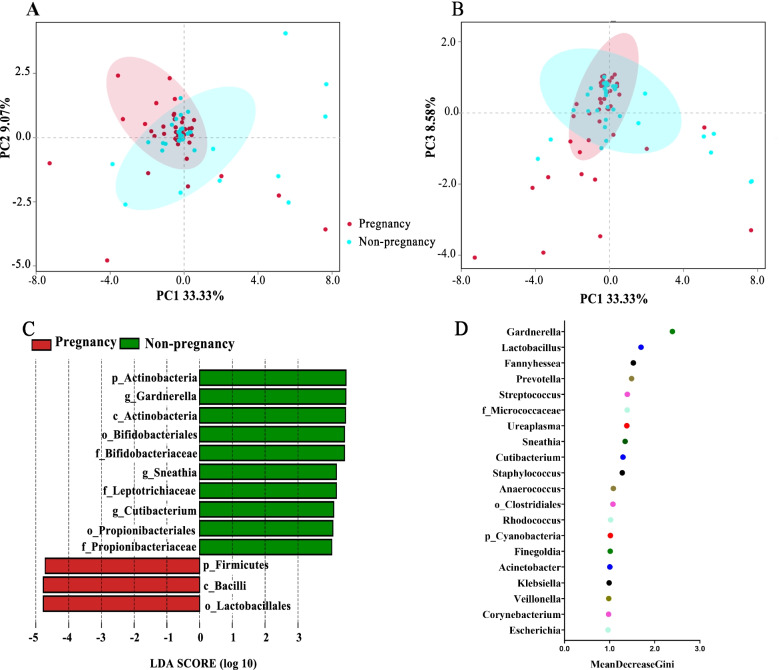
Differences in the vaginal microbiome with different pregnancy outcomes. **A**, **B** PCoA analysis based on Jaccard distance: PC 1, PC2, and PC3 could explain 33.33%, 9.07%, and 8.58% of the variation, respectively. **C** Lefse analysis; the threshold of LDA value was 4.0. **D** The rank of the Gini index from the random forest model. PCoA, principal coordinate analysis; Lefse, linear discriminant analysis effect size

**Fig. 3:**
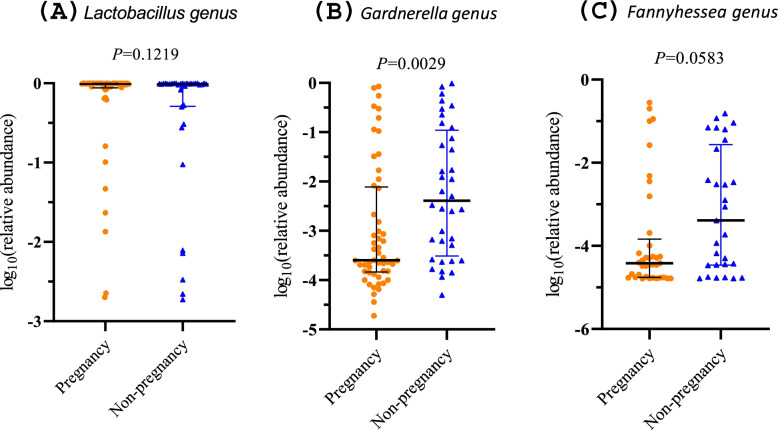
Scatter diagrams showing the relative abundances of the genera between the pregnant and non-pregnant groups. The middle lines represent the median, while the error bars represent the interquartile range. *P* values were acquired by the Kruskal-Wallis test

All nucleic acid samples from vaginal swabs were sequenced successfully, and the sequencing depths were sufficient (Additional file 2: Fig. S1). The sequencing quality of all samples was confirmed to be good (all Q20 indices were > 95%, Additional file 2: Table S2). At the genus level, the most common bacteria with the highest abundances were *Lactobacilli* (mean relative abundance 79.95%), *Gardnerella* (8.57%), S*treptococcus* (1.79%), and *Atopobium* (1.54%) (Additional file 2: Fig. S2). Comparisons of the Shannon, Simpson, Chao1, and ACE indices showed that pre-pregnancy vaginal bacterial diversities were not statistically different when compared between the pregnant and non-pregnant groups (*P* > 0.05, Additional file 2: Fig. S3). However, PCoA showed that the vaginal microbial community structures of these two groups were slightly different; 2.5% of variations were associated with pregnancy outcomes (PERMANOVA test, *R*^2^ = 0.025, *P* = 0.049, Fig. 2A, B). To further identify the key species, we performed a Lefse analysis; the results showed that the abundance of the *Lactobacillales* order in the pregnant group was higher than that in the non-pregnant group (LDA > 4.0, average relative abundance 86.33% vs 75.63%). The abundance of the *Actinobacteria* phylum (8.64% vs 14.01%, LDA > 4.0) and the *Gardnerella* genus (6.34% vs 11.84%, LDA > 4.0) in the non-pregnant group was higher than those in the pregnant group (Fig. 2C). At the genus level, random forest model analysis identified potential biomarkers for distinguishing pregnancy or non-pregnancy, including *Gardnerella*, *Lactobacillus*, and *Fannyhessea*, with a relatively high Gini index (Fig. 2D). Based on these results, we further compared the relative abundance of this genus among the two groups (Fig. 3). The relative abundance of *Gardnerella* in the pregnant group was significantly lower than that in the non-pregnant group (*P* = 0.0029).

### Association validation in phase II

Based on the findings from phase I, we further focused on the *Gardnerella*, *Fannyhessea*, and *Lactobacillus* genera. In consideration of the leading role of *Lactobacillus* in the vaginal microbiome, and the potentially differential effects of different *Lactobacillus species*, except for the *G. vaginalis* and *F. vaginae*, we also detected the three most common *Lactobacillus* species, including *L. cripatus*, *L. iners*, and *L. gassri*. In total, 332 women were included in the phase II analysis. The mean age was 29.50 ± 3.95 years,, and the mean age difference within couples was 1.35 years. The baseline characteristics of women who were excluded for various reasons were comparable with those included (Additional file 2: Table S3). The absolute loads of *L. crispatus*, *L. gasseri*, *L. iners*, *G. vaginalis*, and *F. vaginae* in the vaginal swabs taken at baseline were detected using standard curves (Additional file 2: Fig. S4). The correlation analysis shows that *L. crispatus* was positively associated with *L. gasseri* (*ρ* = 0.56, *P* < 0.001) and negatively associated with *L. iners* (*ρ* = 0.18, *P =* 0.004). *L. iners* was negatively associated with *L. gasseri* (*ρ* = − 0.14, *P* = 0.009). *G. vaginalis* was positively associated with *F. vaginae* (*ρ* = 0.31, *P* < 0.001). The associations between the remaining species were not statistically significant (*P* > 0.05), the details were shown in Additional file 2: Table S4.

The absolute loads of these species at baseline were then divided into four groups (Q1–Q4) based on the interquartile range. Cox models were then used to estimate the association between these four groups and female fecundability (Additional file 2: Table S5). Data showed that female fecundability increased with higher absolute loads of *L. gasseri* (Q4 vs Q1, FR = 1.71, 95%CI 1.02–2.87) but decreased with higher absolute loads of *F. vaginae* (Q4 vs Q1, FR = 0.62, 95%CI 0.38–1.00). Other species were not statistically associated with female fecundability (*P* > 0.05).

### Vaginal microbiome type and fecundability

**Fig. 4:**
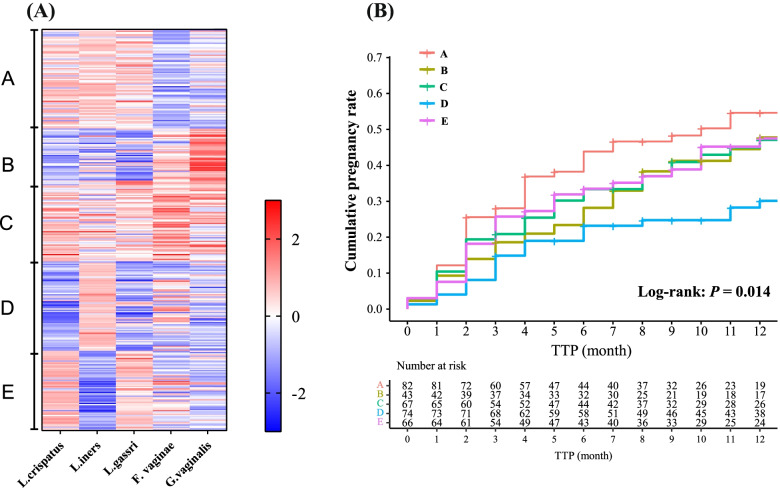
Types of vaginal microbiome and fecundability. **A** Clustering analysis based on the absolute loads of five bacterial species, based on the *z*-score of the log_10_(absolute load). **B** Kaplan-Meier plots for the cumulative pregnancy rate across different vaginal microbiome types

Based on the absolute loads of five bacterial species, we found that the vaginal microbiome clustered into five types (A–E). Figure 4A shows that type A (24.7%, 82/332) featured three high abundant *Lactobacillus* species, with a low abundance of *G. vaginalis* and *F. vaginae*. Type B (13.0%, 43/332) was characterized by a high abundance of *G. vaginalis* and a low abundance of the three *Lactobacillus* species. Type C (20.2%, 67/332) was characterized by a high abundance of *F. vaginae* and a modest abundance of the three *Lactobacillus* species. Type D (22.3%, 74/332) was characterized by a high abundance of *L. iners* abundance and low abundances of the other four species. Type E (19.9%, 66/332) was characterized by high abundances of *L. crispatus* and *L. gasseri* and low abundances of the other three species. The *z*-scores of the absolute abundance of specific species grouped by different types are shown in Additional file 2: Table S6. Figure 4B showed that women with different types of vaginal microbiome had different levels of fecundability (log-rank test, *P* = 0.014). Women with the type A vaginal microbiome had the highest cumulative pregnancy rate (12th month, 54.7%, 95%CI 41.2–65.1%) while women with the type D vaginal microbiome had the lowest cumulative pregnancy rate (12th month, 28.2%, 95%CI 16.8–38.1%). Types B, C, and E had similar cumulative pregnancy rates (12th month, 44.5% vs 45.0% vs 45.2%).

After adjusting for potential confounding factors, including female age, educational level, occupation, pregnancy history, vaginal cleanliness grading, and the age difference between couples, we found that compared to women with a type A vaginal microbiome, women with a type D microbiome showed a 55% reduction in fecundability (model A, FR = 0.45, 95%CI 0.26–0.76). This association was robust irrespective of whether the TTP was determined by month or menstrual cycle (model B, FR = 0.45, 95%CI 0.27–0.77). Women with vaginal microbiome types B, C, and E had lower tendencies of fecundability compared with type A, although these differences were not statistically significant (Table 2). Sensitivity analysis based on the dataset of completed cases was consistent with these primary results (Additional file 2: Table S7).

**Table 2:** Fecundability ratios for different vaginal microbiome types

Type	*N* (%)	Crude FR (95%CI)	Model A, FR (95%CI)	Model B, FR (95%CI)
A	82 (24.7)	Ref	Ref	Ref
B	43 (13.0)	0.75 [0.45, 1.26]	0.84 [0.48, 1.46]	0.86 [0.49, 1.50]
C	67 (20.2)	0.70 [0.44, 1.12]	0.81 [0.51, 1.30]	0.82 [0.51, 1.31]
D	74 (22.3)	0.41 [0.24, 0.68]*	0.45 [0.26, 0.76]*	0.45 [0.27, 0.77]*
E	66 (19.9)	0.70 [0.44, 1.11]	0.71 [0.44, 1.15]	0.71 [0.44, 1.14]

Model A: TTP was determined by month. FRs were adjusted for female age, the age difference between couples, educational level, occupation, pregnancy history, and vaginal cleanliness grading

Model B: TTP was determined by the menstrual cycle. FRs were adjusted for female age, the age difference between couples, educational level, occupation, pregnancy history, and vaginal cleanliness grading

*The FR is statistically significant

## Discussion

Our two-stage cohort study aimed to demonstrate the association between the pre-pregnancy vaginal microbiome and female fecundability among healthy pregnancy-planning women. The results supported this association and suggested that the higher relative abundances of *L. crispatus* and *L. gasseri* were positively associated with female fecundability, while a higher relative abundance of *F. vaginae* appeared to be detrimental to female fecundability. From a community perspective, a vaginal microbiome characterized by a higher abundance of *L. iners* and *a* lower abundance of *L. crispatus* and *L. gasseri* appears to be associated with a lower fecundability. This study provides more credible evidence than previous studies in that we demonstrated that it is possible to predict female fecundability by assessing the pre-pregnancy vaginal microbiome.

Many studies have focused on the damaging effects of BV on infertility and, in particular, tubal infertility. However, the case-controlled design of these studies limited causal inference [4]. It is difficult to collect vaginal swabs before a patient is diagnosed as being infertile. Furthermore, the precise role played by the vaginal microbiome in cases of non-tubal infertility, and in particular, unexplained infertility, remains unknown [25]. While some studies found that women with a better vaginal environment appeared to have a higher chance of successful embryo implantation when undergoing in vitro fertilization (IVF) [26], a recent meta-analysis did not identify a significant impact of BV on the live birth rate or clinical pregnancy rate in women undergoing IVF [27]. Thus far, the screening and treatment of BV before attempting conception remain a possibility but are not a widely accepted consensus [28]. A Kenyan cohort study provided a clue that BV appeared to be negatively associated with female fecundability [6]; however, the microscopy-based vaginal microenvironment assessment could not fully reflect the status of the vaginal microbiota, especially considering the diverse effects of different *Lactobacillus* spp. [8]. Next-generation sequencing technology provides an opportunity to explain many unknown problems [29]. A retrospective case-control study, with a small sample size, revealed that major vaginal microbiota clusters could not be grouped by infertility status [30]. The present, prospective study is the first to demonstrate the different effects of *L. crispatus*, *L. gasseri*, and *L. iners*, on female fecundability.

*Lactobacillus* has always been regarded as a biomarker for a healthy vaginal microenvironment. One of the most important reasons for this is that this species can produce lactic acid to maintain a locally acidic environment to prevent pathogen colonization [31]. Local inflammation, caused by disordered vaginal microbiota, may lead to reduced levels of fertility; higher levels of cervical interleukin (IL)-1b, IL-6, and IL-8 cytokines have been reported to be associated with infertility [32]. *L. iners* was also shown to produce a type of protein toxin (inerolysin) that might play a potential role in the pathogenesis of bacterial vaginosis [33]. This mechanism might explain our current findings in that the vaginal microbiome characterized by a higher abundance of *L. iners* and a lower abundance of *L. crispatus* and *L. gasseri* is the only type that could reduce fecundability when compared with other microbiome types. However, studying the effects of individual *L. iners* seems less important than studying the effects of the microbiome as a whole. Our study found a type A microbiome (characterized by three *Lactobacillus* species, including *L. iners*) was the best type for fecundability. Thus, a comprehensive assessment of vaginal microbial structure seems necessary, especially with regard to different *Lactobacillus* species. Furthermore, Li et al. demonstrated that the vaginal probiotic *L. crispatus* greatly affected sperm activity and could also reduce pregnancies via its adhesive properties; this might account for some cases of unexplained infertility [34]. In the present study, we identified the positive effect of *L. crispatus* on fecundability; this suggests that it is important to investigate the dual role of *Lactobacillus* in future research.

*G. vaginalis* and *F. vaginae* have always been regarded as BV-related bacteria [35]; however, the direct association between these species and female fecundability remains unknown. Recent molecular analyses of protein-coding genes demonstrated that *G. vaginalis* consists of at least four distinct sub-species, although not all of these sub-species cause clinical symptoms [36]. Thus, an asymptomatic carrier of *G. vaginalis* might be a potential reason for unexplained infertility. Meanwhile, the presence of *F. vaginae* would lead to the creation of biofilms in the vagina and would resist some antimicrobial substances [37]; however, the effects of these biofilms on sperm motility have yet to be investigated.

Our study was strengthened by the two-stage cohort design. Although many statistical efforts had been carried out, it is possible that the omics study may have led to false positives [38]. Thus, the mutual verification of the results from our two phases increased the robustness of our findings. Compared with a register-based cohort [5], our refined cohort guaranteed the accuracy of TTP estimation. In addition, our novel strategy for defining the vaginal microbiome type provides a new concept for studying the vaginal microflora in the future. However, our study was also associated with some limitations that need to be considered. First, it was very difficult to collect data relating to sperm quality from the couples who were planning pregnancy; this is a vital confounding factor for pregnancy outcome. This potential confounding effect is a critical problem that needs to be solved in future research. Secondly, the sample size was insufficient in phase II, especially when investigating new types of vaginal microbiome; several types showed a decreasing trend for cumulative pregnancy rate, but without statistical significance. Thirdly, the vaginal microbiota appears to change dynamically with menstruation [39]; one sampling event is not able to fully reflect the characteristics of the vaginal microbiota. Fourthly, all of the samples were obtained from a single center; this could influence the extrapolation of our conclusions, especially when considering the variation of vaginal microbiota across different races [40]. Finally, we just only focused on three genera in phase II; further studies should focus on other potential bacteria species, in order to gain a more comprehensive understanding of vaginal microbiome.

## Conclusions

This cohort study demonstrated an association between the pre-pregnancy vaginal microbiome and female fecundability. A vaginal microbiome characterized by a higher abundance of *L. iners* and a lower abundance of *L. crispatus* and *L. gasseri* appears to be associated with a lower fecundability. Further research now needs to confirm whether manipulation of the vaginal microenvironment might improve human fecundability.

## Supplementary Information

**Additional file 1.** Supplementary information.

**Additional file 2: Table S1.** The specific primers used for qPCR. **Table S2.** The sequencing quality for all samples. **Table S3.** The baseline characteristics for the women included and excluded in Phase II. **Table S4.** Spearman’s correlation coefficients between different species. **Table S5.** Fecundability ratios for the absolute loads of different species. **Table S6.** Z scores for the absolute abundance of species grouped by cluster A~E. **Table S7.** Fecundability ratios for different vaginal microbiome types based on a complete case dataset. **Fig. S1.** Rarefaction curves for OTU number. **Fig. S2.** Histogram showing the relative abundance of different genera. **Fig. S3.** Scatter diagram showing different α diversities between pregnancy and non-pregnancy groups. **Fig. S4.** Standard curves for the detection of different species by qPCR.

## Data Availability

The raw sequencing data is stored in the figshare platform: Hong X and Wang B. The raw data for the analysis of the association between the pre-pregnancy vaginal microbiome and time-to-pregnancy. figshare 2022:10.6084/m6089.figshare.20045855.v20045851

## Abbreviations

BH: Benjamini-Hochberg
BV: Bacterial vaginosis
CI: Confidence intervals
*F. vaginae*: *Fannyhessea vaginae*
FRs: Fecundability ratios
*G. vaginalis*: *Gardnerella vaginalis*
IVF: In vitro fertilization
KM: Kaplan-Meier
*L. crispatus*: *Lactobacillus crispatus*
*L. gasseri*: *Lactobacillus gasseri*
*L. iners*: *Lactobacillus iners*
LDA: Linear discriminant analysis effect size
LMP: Last menstrual period
MICE: Multivariate imputation chained equations
NCBI: National Center for Biotechnology Information
OTU: Operational taxonomic unit
PCoA: Principal coordinate analysis
PERMANOVA: Permutational multivariate analysis of variance
qPCR: Quantitative real-time polymerase chain reaction
SD: Standard deviation
TTP: Time-to-pregnancy
