# Supplementary material for: The association between the pre-pregnancy vaginal microbiome and time-to-pregnancy: a Chinese pregnancy-planning cohort study
Source: BMC Med. 2022 Aug 1;20:246. doi: 10.1186/s12916-022-02437-7 (PMC9341075; doi:10.1186/s12916-022-02437-7)
Supplement: Supplementary file 1 — Additional file 1. Supplementary information. [file 12916_2022_2437_MOESM1_ESM.docx]

**Additional file 1**

***Sample size estimations***

In Phase I, we would compare the vaginal microbiome between pregnancy and non-pregnancy women using PERMANOVA test. Through Kelly’s method,[12] we firstly hypothesized that the coefficient of determination between groups (omega-squared) was 0.04, then the *micropower* package was used to estimate the minimum sample size when the statistical power was more than 90%. It needs at least 30 people in each group. We hypothesized that there would be 50% of participants get pregnancy in one year, then almost 20% of participants would lose follow-up, the finial sample size was 30÷0.5÷(1-0.2)=75. So we decided to invite 100 couples in the Phase I. Although such, we invited all the couples took part in the program in one day to the same Phase I, so there were 106 couples were invited finally. The Phase II is a validation for Phase I. After discussion, we decided to include more than 3 times of participants than Phase I, so there were at least 300 couples included in the Phase II.

***Nucleic acids extraction for vaginal swabs***

Swabs were thawed and placed in one ml PBS buffer solution. After 10 min oscillate (Water-bathing Constant Temperature Vibrator, Guohua Machinery Co., Ltd., Jiangsu,China), swabs were removed and the PBS eluates centrifuged for 2 min (12,000 rpm, Centrifuge 5424 R; Eppendorf Co., Ltd., Hamburg, Germany). Sediments were DNA extracted using TIANamp Bacteria DNA Kit (Tiangen Biochemical Technology, Beijing, China), following manufacturer’s instructions. Purified nucleic acids were eluted in TE buffer and quantified on a Nanodrop 2000 (Thermo Fisher Scientific). The hyper-variable V3-V4 region of the 16S rRNA gene was amplified by PCR, using modified 338F (5’-ACTCCTACGGGAGGCAGCA-3’) and 806R (5’-GGACTACHVGGGTWTCTAAT-3’) primers with a unique 12 bp barcode, facilitating sequencing on the Illumina HiSeq platform (Illumina, San Diego, CA, USA). PCR reactions, containing 25 ml 2× Premix Taq (Takara Biotechnology, Dalian Co., Ltd., China), one ml each primer (10 mM) and

three ml DNA (20 ng/ml) template in a final volume of 50 µl, were amplified using the following parameters: Initialization for 5 min at 94 ℃; 30 cycles of 30 s denaturation at 94 ℃, 30 s annealing at 52 ℃, and 30 s extension at 72 ℃; followed by a 10 min elongation at 72 ℃. PCR was performed on a BioRad S1000 (Bio-Rad Laboratory, CA, USA). The PCR products were sequenced on an IlluminaHiseq2500 platform.

***Sequencing data processed***

16S rRNA gene sequencing data were merged with FLASH (v1.2.7, http://ccb.jhu.edu/software/FLASH/) and Trimmomatic software (v0.33) was used to remove tags of low quality (with more than six mismatches compared to the primers, average quality score < 20 in a 50 bp sliding window or shorter than 350 bp). Denoised sequences were clustered using USEARCH (version 10.0), and tags with ≥ 97% similarity regarded as an operational taxonomic unit (OTU). Representative sequence were annotated through NCBI dataset using QIIME software. The numbers of reads for each sample were normalized corresponding to the sample with the least sequence.
