# Supplementary material for: The association between the pre-pregnancy vaginal microbiome and time-to-pregnancy: a Chinese pregnancy-planning cohort study
Source: BMC Med. 2022 Aug 1;20:246. doi: 10.1186/s12916-022-02437-7 (PMC9341075; doi:10.1186/s12916-022-02437-7)
Supplement: Supplementary file 2 — Additional file 2: Table S1. The specific primers used for qPCR. Table S2. The sequencing quality for all samples. Table S3. The baseline characteristics for the women included and excluded in Phase II. Table S4. Spearman’s correlation coefficients between different species. Table S5. Fecundability ratios for the absolute loads of different species. Table S6. Z scores for the absolute abundance of species grouped by cluster A~E. Table S7. Fecundability ratios for different vaginal microbiome types based on a complete case dataset. Fig. S1. Rarefaction curves for OTU number. Fig. S2. Histogram showing the relative abundance of different genera. Fig. S3. Scatter diagram showing different α diversities between pregnancy and non-pregnancy groups. Fig. S4. Standard curves for the detection of different species by qPCR. [file 12916_2022_2437_MOESM2_ESM.docx]

**Additional file 2 contents**

Table S1. The specific primers used for qPCR.

Table S2: The sequencing quality for all samples

Table S3. The baseline characteristics for the women included and excluded in Phase II

Table S4. Spearman’s correlation coefficients between different species.

Table S5. Fecundability ratios for the absolute loads of different species.

Table S6. Z scores for the absolute abundance of species grouped by cluster A~E

Table S7. Fecundability ratios for different vaginal microbiome types based on a complete case dataset.

Figure S1. Rarefaction curves for OTU number

Figure S2. Histogram showing the relative abundance of different genera.

Figure S3. Scatter diagram showing different α diversities between pregnancy and non-pregnancy groups.

Figure S4. Standard curves for the detection of different species by qPCR.

**Table S1. The specific primers used for qPCR.**

| Species | Primers | Length | Reference |
| --- | --- | --- | --- |
| *L. crispatus* | F- Primer: 5’-AGCGAGCGGAACTAACAGATTTAC-3’  R-Primer: 5’-AGCTGATCATGCGATCTGCTT-3’ | 154bp | 18 |
| *L.iners* | F-Primer: 5’-GTC TGC CTT GAA GAT CGG-3’  R-Primer: 5’-ACA GTT GAT AGG CAT CAT C-3’ | 158bp | 19 |
| *L. gasseri* | F-Primer: 5’-TGGAAACAGRTGCTAATACCG-3’  R-Primer: 5’-CAGTTACTACCTCTATC  TTTCTTCACTAC-3’ | 322bp | 18 |
| *G. vaginalis* | F-Primer：5’-TTAC TGGT GTAT CACT GGTA AG-3’  R-Primer：5’-CCGT CAGA GGCT GAAC AGT-3’ | 330bp | 20 |
| *A. vaginae* | F-Primer：5’-GGTGAAGCAGTGGAAACACT-3’  R-Primer：5’-ATTCGCTTCTGCTCGCGCA-3’ | 277bp | 19 |
| 16S | F-Primer：5’-ACTCCTACGGGAGGCAGCA-3’  R-Primer：5’-GGACTACHVGGGTWTCTAAT-3’ | 469bp | 21 |

**Table S2: The sequencing quality for all samples**

| **Sample ID** | **Raw Reads** | **Clean Reads** | **Effective Reads** | **AvgLen(bp)** | **GC(%)** | **Q20(%)** | **Q30(%)** | **Effective(%)** |
| --- | --- | --- | --- | --- | --- | --- | --- | --- |
| 1 | 80087 | 60010 | 59993 | 429 | 51.23 | 98.02 | 96.29 | 74.91 |
| 2 | 79965 | 56986 | 56939 | 429 | 51.71 | 97.99 | 96.14 | 71.2 |
| 3 | 79680 | 53299 | 51395 | 415 | 53.1 | 98.3 | 96.45 | 64.5 |
| 4 | 80097 | 55547 | 53760 | 423 | 53.17 | 98.18 | 96.36 | 67.12 |
| 5 | 80109 | 61695 | 60874 | 429 | 51.27 | 98.09 | 96.34 | 75.99 |
| 6 | 80078 | 61722 | 61466 | 429 | 51.05 | 98.08 | 96.31 | 76.76 |
| 7 | 79884 | 60845 | 60832 | 429 | 51 | 98.03 | 96.29 | 76.15 |
| 8 | 79841 | 59812 | 59496 | 429 | 51.04 | 98.06 | 96.31 | 74.52 |
| 9 | 80059 | 58836 | 58209 | 429 | 51.23 | 98.06 | 96.33 | 72.71 |
| 10 | 80064 | 57463 | 56105 | 429 | 51.69 | 98.05 | 96.25 | 70.08 |
| 11 | 79838 | 52925 | 52468 | 429 | 51.94 | 98.08 | 96.27 | 65.72 |
| 12 | 80095 | 57907 | 56947 | 429 | 51.59 | 98.03 | 96.21 | 71.1 |
| 13 | 79800 | 60844 | 59083 | 429 | 51.35 | 98.04 | 96.23 | 74.04 |
| 14 | 79854 | 53291 | 52655 | 412 | 57.21 | 98.35 | 96.43 | 65.94 |
| 15 | 79995 | 60148 | 59307 | 429 | 51.29 | 98.01 | 96.22 | 74.14 |
| 16 | 80099 | 60556 | 60543 | 429 | 51.67 | 98.13 | 96.36 | 75.59 |
| 17 | 80302 | 61414 | 61397 | 429 | 51.06 | 98.06 | 96.35 | 76.46 |
| 18 | 80084 | 61423 | 61358 | 429 | 51.26 | 98.08 | 96.37 | 76.62 |
| 19 | 80307 | 62334 | 62010 | 429 | 51.29 | 98.13 | 96.44 | 77.22 |
| 20 | 80097 | 60300 | 59919 | 429 | 50.82 | 98.03 | 96.27 | 74.81 |
| 21 | 80031 | 60905 | 60721 | 428 | 51.13 | 98.11 | 96.34 | 75.87 |
| 22 | 79759 | 61148 | 61085 | 429 | 51 | 98.02 | 96.28 | 76.59 |
| 23 | 80183 | 61388 | 61341 | 429 | 51.2 | 98.08 | 96.36 | 76.5 |
| 24 | 79954 | 54012 | 50908 | 416 | 51.09 | 98.23 | 96.39 | 63.67 |
| 25 | 80109 | 52617 | 50171 | 417 | 53.33 | 98.25 | 96.37 | 62.63 |
| 26 | 79735 | 54224 | 51027 | 416 | 50.98 | 98.2 | 96.36 | 64 |
| 27 | 66191 | 45209 | 45097 | 418 | 54.01 | 98.23 | 96.35 | 68.13 |
| 28 | 80222 | 53438 | 52421 | 414 | 56.29 | 98.27 | 96.38 | 65.34 |
| 29 | 79843 | 54966 | 54925 | 412 | 57.45 | 98.44 | 96.6 | 68.79 |
| 30 | 79990 | 61705 | 61656 | 429 | 51.22 | 98.1 | 96.38 | 77.08 |
| 31 | 80104 | 58353 | 56737 | 427 | 51.96 | 98.05 | 96.2 | 70.83 |
| 32 | 80002 | 59475 | 59306 | 429 | 51.92 | 98.12 | 96.34 | 74.13 |
| 33 | 80223 | 59183 | 57971 | 429 | 51.57 | 98.06 | 96.27 | 72.26 |
| 34 | 79939 | 58826 | 57913 | 429 | 51.56 | 98.04 | 96.25 | 72.45 |
| 35 | 79777 | 54354 | 53670 | 422 | 54.22 | 98.08 | 96.23 | 67.28 |
| 36 | 80200 | 60439 | 60295 | 429 | 51.29 | 98.04 | 96.31 | 75.18 |
| 37 | 80158 | 62113 | 62018 | 429 | 51.18 | 98.1 | 96.37 | 77.37 |
| 38 | 80307 | 59881 | 58999 | 429 | 51.69 | 98.07 | 96.29 | 73.47 |
| 39 | 80125 | 60630 | 60009 | 428 | 51.68 | 98.09 | 96.33 | 74.89 |
| 40 | 79950 | 59426 | 59057 | 429 | 51.78 | 98.06 | 96.26 | 73.87 |
| 41 | 80080 | 55772 | 54348 | 415 | 56 | 98.31 | 96.45 | 67.87 |
| 42 | 80010 | 62494 | 62414 | 429 | 51.24 | 98.09 | 96.35 | 78.01 |
| 43 | 79826 | 60545 | 59699 | 429 | 51.58 | 98.07 | 96.31 | 74.79 |
| 44 | 79751 | 56283 | 54691 | 422 | 54.05 | 98.14 | 96.26 | 68.58 |
| 45 | 80110 | 59649 | 59288 | 427 | 52.11 | 98.12 | 96.33 | 74.01 |
| 46 | 80103 | 60457 | 59322 | 429 | 51.48 | 98.07 | 96.3 | 74.06 |
| 47 | 80083 | 60803 | 60756 | 429 | 51.67 | 98.1 | 96.32 | 75.87 |
| 48 | 79977 | 60466 | 60380 | 429 | 51.27 | 98.02 | 96.28 | 75.5 |
| 49 | 70252 | 50425 | 49735 | 429 | 51.15 | 98.06 | 96.26 | 70.8 |
| 50 | 79366 | 58510 | 57071 | 429 | 51.69 | 98.05 | 96.22 | 71.91 |
| 51 | 79984 | 57475 | 56870 | 429 | 51.49 | 97.98 | 96.13 | 71.1 |
| 52 | 79757 | 56865 | 56402 | 428 | 52.29 | 98.05 | 96.21 | 70.72 |
| 53 | 79784 | 60867 | 60769 | 429 | 51.22 | 98.05 | 96.31 | 76.17 |
| 54 | 79786 | 57026 | 56659 | 429 | 51.62 | 98 | 96.16 | 71.01 |
| 55 | 79662 | 60048 | 59888 | 429 | 51.18 | 98.05 | 96.29 | 75.18 |
| 56 | 79998 | 56735 | 56125 | 424 | 53.47 | 98.11 | 96.23 | 70.16 |
| 57 | 80006 | 59682 | 59669 | 429 | 51.66 | 98.06 | 96.25 | 74.58 |
| 58 | 79924 | 58556 | 58535 | 429 | 51.64 | 98.08 | 96.3 | 73.24 |
| 59 | 79755 | 53253 | 51231 | 417 | 54.47 | 98.3 | 96.41 | 64.24 |
| 60 | 80073 | 60274 | 59788 | 429 | 51.5 | 98.13 | 96.39 | 74.67 |
| 61 | 80231 | 56818 | 56589 | 426 | 52.62 | 98.06 | 96.2 | 70.53 |
| 62 | 80256 | 60370 | 60239 | 429 | 51.64 | 98.14 | 96.33 | 75.06 |
| 63 | 79945 | 60755 | 60675 | 429 | 51.24 | 98.1 | 96.4 | 75.9 |
| 64 | 80216 | 59124 | 58103 | 429 | 51.52 | 98.03 | 96.22 | 72.43 |
| 65 | 80098 | 59468 | 59374 | 429 | 51.24 | 98.01 | 96.27 | 74.13 |
| 66 | 79981 | 59578 | 59409 | 429 | 51.24 | 98.01 | 96.25 | 74.28 |
| 67 | 80073 | 61208 | 61067 | 429 | 51.32 | 98.05 | 96.32 | 76.26 |
| 68 | 79775 | 58088 | 57143 | 429 | 51.48 | 98.03 | 96.22 | 71.63 |
| 69 | 80222 | 56676 | 56181 | 422 | 53.04 | 98.01 | 96.07 | 70.03 |
| 70 | 79863 | 59509 | 59328 | 429 | 50.97 | 98.02 | 96.24 | 74.29 |
| 71 | 80336 | 59814 | 59420 | 429 | 51.28 | 97.96 | 96.14 | 73.96 |
| 72 | 79851 | 52906 | 52062 | 412 | 57.37 | 98.4 | 96.53 | 65.2 |
| 73 | 79954 | 59435 | 59051 | 429 | 51.08 | 98.05 | 96.28 | 73.86 |
| 74 | 80398 | 60819 | 60609 | 429 | 51.18 | 98.08 | 96.36 | 75.39 |
| 75 | 80163 | 61722 | 61590 | 429 | 51.22 | 98.1 | 96.39 | 76.83 |
| 76 | 79901 | 61426 | 61108 | 429 | 51.23 | 98.07 | 96.31 | 76.48 |
| 77 | 80027 | 57835 | 57768 | 429 | 51.72 | 98.05 | 96.23 | 72.19 |
| 78 | 80141 | 60644 | 59811 | 429 | 51.44 | 98.14 | 96.42 | 74.63 |
| 79 | 80025 | 56057 | 54486 | 422 | 50.3 | 98.15 | 96.35 | 68.09 |
| 80 | 79904 | 58964 | 57611 | 427 | 51.37 | 98.14 | 96.42 | 72.1 |
| 81 | 80006 | 60831 | 60450 | 429 | 51.05 | 98.13 | 96.4 | 75.56 |
| 82 | 79933 | 61086 | 60916 | 429 | 51.11 | 98.09 | 96.37 | 76.21 |
| 83 | 79778 | 61842 | 61791 | 429 | 51.66 | 98.18 | 96.41 | 77.45 |
| 84 | 80047 | 60358 | 59400 | 428 | 51.71 | 98.11 | 96.33 | 74.21 |
| 85 | 80017 | 61143 | 60852 | 429 | 51.32 | 98.07 | 96.32 | 76.05 |
| 86 | 79937 | 60942 | 60898 | 429 | 51.65 | 98.13 | 96.34 | 76.18 |
| 87 | 80201 | 54260 | 52367 | 415 | 54.15 | 98.34 | 96.5 | 65.29 |
| 88 | 80009 | 60382 | 60366 | 429 | 51.66 | 98.13 | 96.36 | 75.45 |
| 89 | 80067 | 61336 | 60330 | 426 | 50.54 | 98.15 | 96.4 | 75.35 |

**Table S3. The baseline characteristics for the women included and excluded in Phase II**

|  | Included  N=332 | Excluded ^a^  N=57 | χ^2^/*t* | *P* |
| --- | --- | --- | --- | --- |
| Age, y, mean (SD) | 29.50 (3.95) | 29.18 (4.71) | 0.49 | 0.629 |
| Age difference with their couples, y, mean (SD) | 1.35 (2.86) | 1.44 (3.99) | 0.16 | 0.875 |
| Educational level |  |  | 3.49 | 0.061 |
| High school and below | 44 (13.4) | 2 ( 3.6) |  |  |
| Higher education and above | 285 (86.6) | 54 (96.4) |  |  |
| Occupation |  |  | 1.25 | 0.534 |
| Workers | 4 ( 1.2) | 1 ( 1.9) |  |  |
| Office clerk | 233 (72.8) | 34 (65.4) |  |  |
| Others | 83 (25.9) | 17 (32.7) |  |  |
| Pregnancy history |  |  | 0.78 | 0.377 |
| No | 243 (73.6) | 38 (80.9) |  |  |
| Yes | 87 (26.4) | 9 (19.1) |  |  |
| Regular menstruation |  |  | <0.001 | >0.999 |
| Yes | 45 (14.1) | 6 (14.3) |  |  |
| No | 274 (85.9) | 36 (85.7) |  |  |

Note：a. The women were excluded for without vaginal swabs (19) and withdrawing before first visit (38)

b. Missing data count: educational level (4)；occupation (17); pregnancy history (12)；regular menstruation (28)

**Table S4. Spearman’s correlation coefficients between different species.** (In parentheses are P values).

| Species | Log_10_ (copies/ml)  Median (range interquartile) | Spearman rho | | | | |
| --- | --- | --- | --- | --- | --- | --- |
|  |  | *L. crispatus* | *L. iners* | *L. gasseri* | *G. vaginalis* | *A. vaginae* |
| *L. crispatus* | 5.65 (3.21-7.23) | NA | **-0.18 (0.004)** | **0.56 (<0.001)** | -0.07 (0.140) | 0.03 (0.803) |
| *L. iners* | 7.07 (4.82-7.84) |  | NA | **-0.14^*^ (0.009)** | -0.10 (0.501) | -0.07 (0.130) |
| *L. gasseri* | 4.96 (3.20-5.89) |  |  | NA | -0.03 (0.609) | -0.06 (0.844) |
| *G. vaginalis* | 5.27 (4.02-7.34) |  |  |  | NA | **0.31^**^ (<0.001)** |
| *F. vaginae* | 7.36 (6.03-8.62) |  |  |  |  | NA |

* *P*<0.05；** *P*<0.001

**Table S5. Fecundability ratios for the absolute loads of different species.**

| Species | Crude FR（95%CI） | Model A  FR（95%CI） | Model B  FR（95%CI） |
| --- | --- | --- | --- |
| ***L. crispatus*** |  |  |  |
| Continuous | 1.04 [0.97, 1.11] | 1.04 [0.97, 1.12] | 1.03 [0.96, 1.11] |
| Q1 | Ref | Ref | Ref |
| Q2 | 0.98 [0.61, 1.59] | 0.93 [0.57, 1.54] | 0.92 [0.56, 1.51] |
| Q3 | 1.46 [0.93, 2.29] | 1.51 [0.95, 2.41] | 1.47 [0.93, 2.35] |
| Q4 | 1.21 [0.76, 1.93] | 1.20 [0.74, 1.94] | 1.17 [0.72, 1.88] |
| ***L. iners*** | 1.03 [0.95, 1.12] | 1.04 [0.95, 1.13] | 1.03 [0.95, 1.13] |
| Continuous |  |  |  |
| Q1 | Ref | Ref | Ref |
| Q2 | 1.23 [0.77, 1.95] | 1.29 [0.79, 2.08] | 1.29 [0.79, 2.09] |
| Q3 | 1.18 [0.74, 1.86] | 1.18 [0.74, 1.90] | 1.19 [0.75, 1.92] |
| Q4 | 1.09 [0.68, 1.74] | 1.08 [0.66, 1.78] | 1.09 [0.66, 1.79] |
| ***L. gasseri*** |  |  |  |
| Continuous | **1.10 [1.01, 1.21]*** | 1.09 [0.99, 1.20] | 1.09 [0.99, 1.19] |
| Q1 | Ref | Ref |  |
| Q2 | 1.28 [0.79, 2.09] | 1.31 [0.79, 2.18] | 1.30 [0.78, 2.16] |
| Q3 | 1.36 [0.84, 2.21] | 1.51 [0.91, 2.52] | 1.48 [0.89, 2.47] |
| Q4 | **1.87 [1.17, 2.98]*** | **1.72 [1.03, 2.88]*** | **1.71 [1.02, 2.87]*** |
| ***G. vaginalis*** |  |  |  |
| Continuous | 1.05 [0.98, 1.12] | 1.06 [0.99, 1.14] | 1.06 [0.99, 1.14] |
| Q1 | Ref | Ref |  |
| Q2 | 0.95 [0.65, 1.41] | 0.95 [0.63, 1.44] | 1.09 [0.72, 1.64] |
| Q3 | 1.25 [0.83, 1.89] | 1.23 [0.79, 1.92] | 1.22 [0.78, 1.91] |
| Q4 | 0.90 [0.61, 1.34] | 1.01 [0.65, 1.56] | 0.90 [0.58, 1.41] |
| ***A. vaginae*** |  |  |  |
| Continuous | **0.90 [0.81, 0.99]*** | 0.92 [0.83, 1.02] | 0.92 [0.83, 1.02] |
| Q1 | Ref | Ref | Ref |
| Q2 | 1.04 [0.67, 1.60] | 0.91 [0.58, 1.43] | 0.91 [0.58, 1.43] |
| Q3 | 0.84 [0.53, 1.32] | 0.92 [0.58, 1.46] | 0.94 [0.59, 1.49] |
| Q4 | **0.58 [0.36, 0.94]*** | **0.61 [0.37, 1.00]*** | **0.62 [0.38, 1.00]** |

Model A: TTP was counted by month. FRs were adjusting for female age, the age difference between couples, educational level, occupation, pregnancy history.

Model B: TTP was counted by menstrual cycle. FRs were adjusting for female age, the age difference between couples, educational level, occupation, pregnancy history.

* *P*<0.05

**Table S6. Z scores for the absolute abundance of species grouped by cluster A~E** (means ± standard deviation)

|  | *L.crispatus* | *L.iners* | *L.gassri* | *F. vaginae* | *G.vaginalis* |
| --- | --- | --- | --- | --- | --- |
| A | 0.38±0.67 | 0.52±0.42 | 0.42±0.61 | -0.85±0.52 | -0.33±0.69 |
| B | -0.84±0.57 | -0.57±0.93 | -0.92±0.99 | 0.38±0.73 | 1.50±0.62 |
| C | 0.62±0.71 | 0.31±0.86 | 0.53±0.72 | 1.01±0.66 | 0.54±0.80 |
| D | -1.11±0.65 | 0.67±0.42 | -0.91±0.86 | -0.06±0.98 | -0.60±0.71 |
| E | 0.69±0.59 | -1.34±0.62 | 0.57±0.53 | -1.14±0.90 | -0.45±0.69 |
| *P** | <0.001 | <0.001 | <0.001 | <0.001 | <0.001 |

***** P values were from the variance analysis tests.

**Table S7. Fecundability ratios for different vaginal microbiome types based on a complete case dataset.**

| Type | Crude FR（95%CI） | Model A  FR（95%CI） | Model B  FR（95%CI） |
| --- | --- | --- | --- |
| A | Ref | Ref | Ref |
| B | 0.75 [0.45, 1.26] | 0.83 [0.47, 1.46] | 0.86 [0.50, 1.50] |
| C | 0.70 [0.44, 1.12] | 0.82 [0.51, 1.30] | 0.82 [0.51, 1.31] |
| D | **0.41 [0.24, 0.68]*** | **0.44 [0.26, 0.76]*** | **0.45 [0.27, 0.76]*** |
| E | 0.70 [0.44, 1.11] | 0.71 [0.44, 1.16] | 0.70 [0.43, 1.14] |

Model A: TTP was determined by month. FRs were adjusting for female age, the age difference between couples, educational level, occupation, pregnancy history, and vaginal cleanliness grading.

Model B: TTP was determined by menstrual cycle. FRs were adjusting for female age, the age difference between couples, educational level, occupation, pregnancy history, and vaginal cleanliness grading.


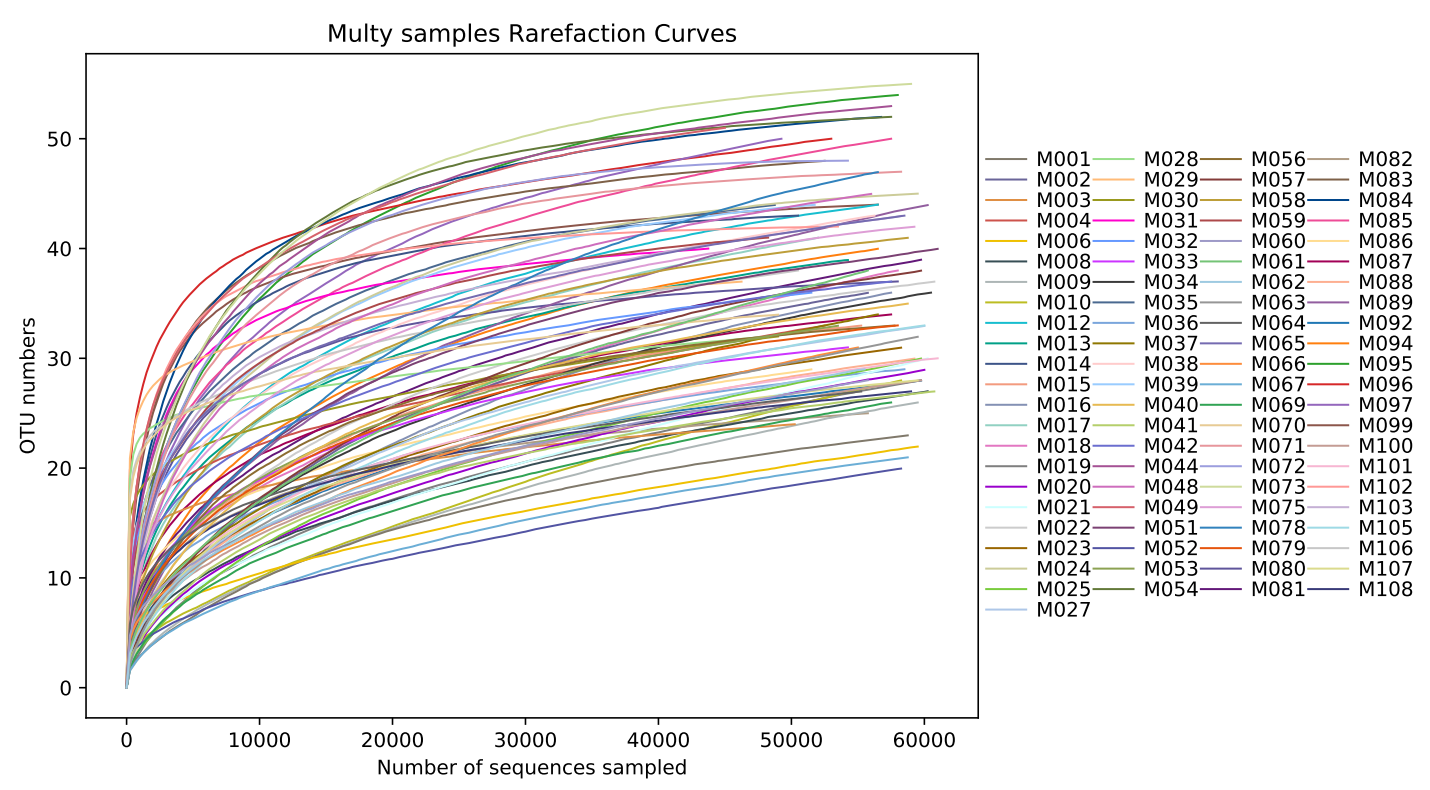


**Figure S1. Rarefaction curves for OTU number**


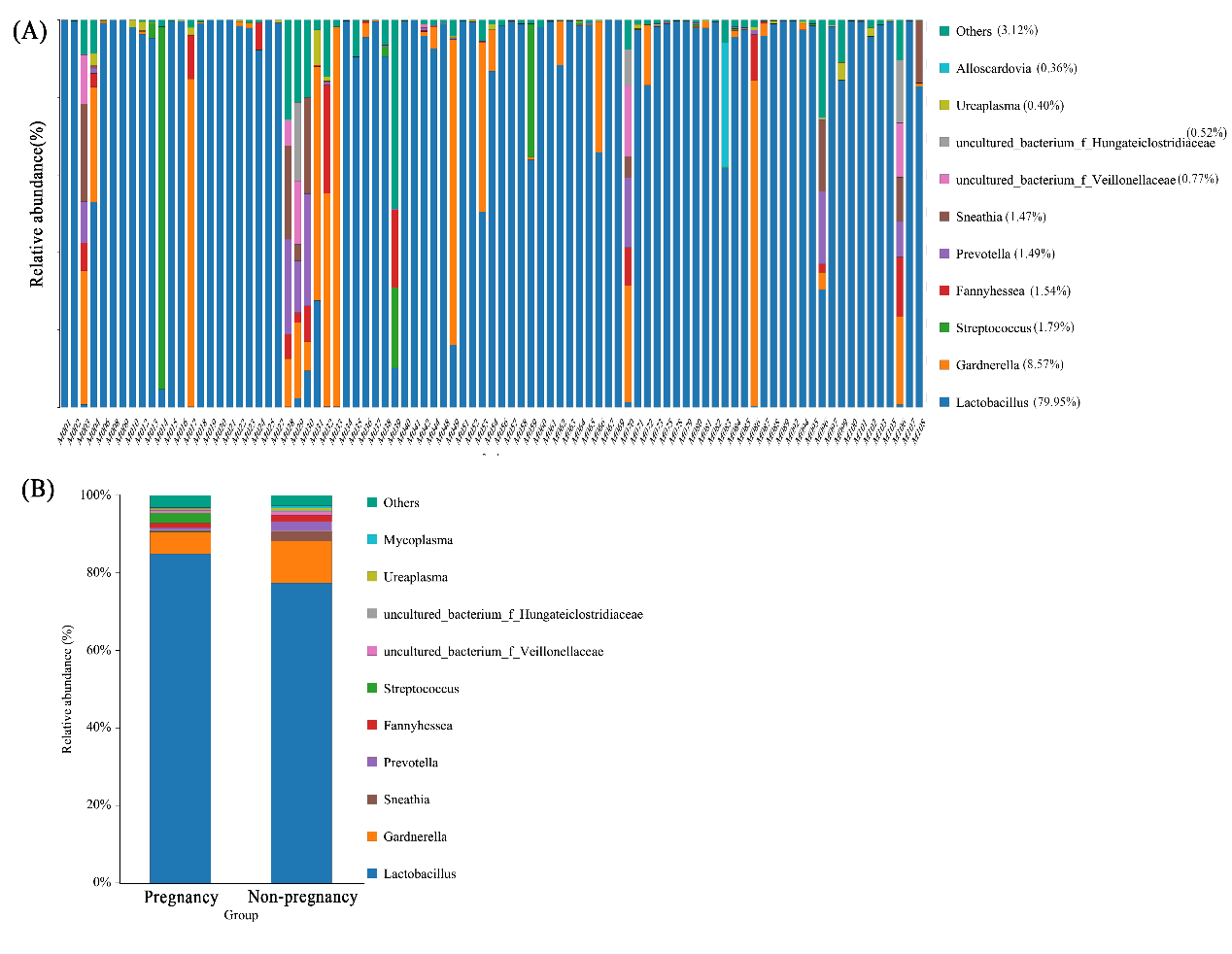


**Figure S2. Histogram showing the relative abundance of different genera.** A) For all the samples. B) for different groups.


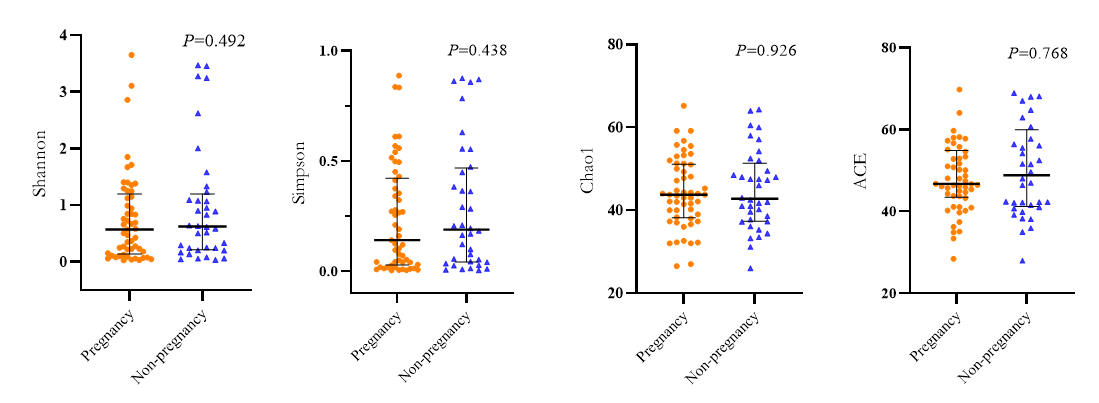


**Figure S3.** **Scatter diagram showing different α diversities between pregnancy and non-pregnancy groups.** The middle lines represent the median, and the error bars represent the range interquartile. *P* values were from Kruskal-Wallis test.


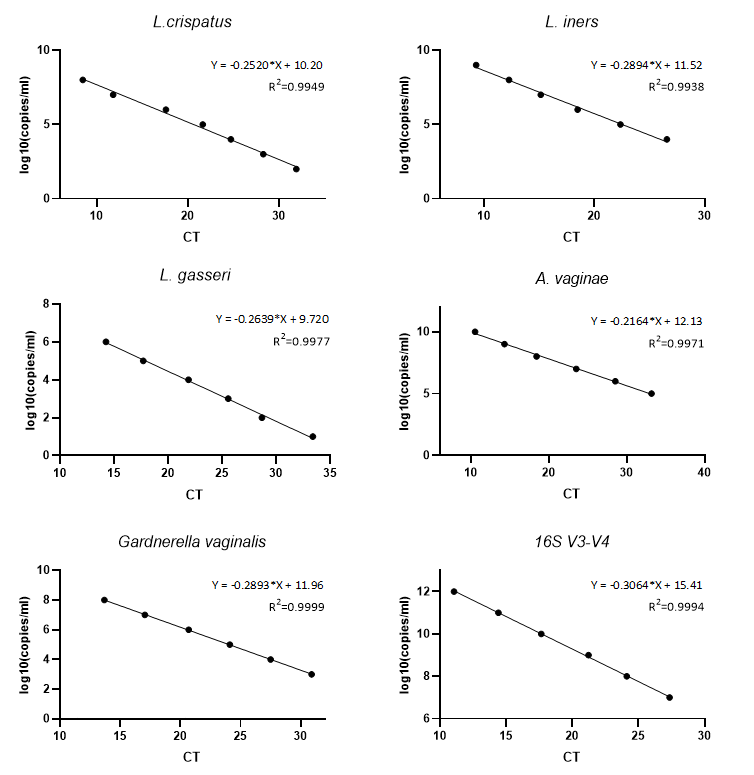


**Figure S4. Standard curves for the detection of different species by qPCR.**
